# Isolated Nasal Tip Metastasis from Esophageal Squamous Cell Carcinoma: Case Report and Literature Review

**DOI:** 10.1155/2015/246094

**Published:** 2015-06-14

**Authors:** Georg J. Ledderose, Anna S. Englhard

**Affiliations:** Department of Otorhinolaryngology, Head and Neck Surgery, Ludwig-Maximilians University of Munich, Marchioninistraße 15, 81377 Munich, Germany

## Abstract

*Objectives*. Cutaneous metastases can be the first sign of a malignant disease and have an unfavorable prognostic significance. The external nose is rarely affected. The uncommon clinical presentation of these cutaneous metastases may lead to the wrong diagnosis and treatment. *Methods*. We present the case of a 59-year-old patient with a small indolent tumor on the tip of the nose that turned out to be the first sign of an extended esophageal cancer. *Conclusion*. The differential diagnosis of tumors of the facial skin and the nasal tip includes metastases from an unknown primary tumor. In rare cases, squamous cell carcinoma of the esophagus needs to be considered.

## 1. Introduction

The differential diagnosis of tumors of the external nose and the nasal vestibule is extensive. It includes local infections, benign tumors like fibromas, chondromas, cysts, hemangiomas, rosacea, and rhinophyma, as well as granulomatous changes occurring in tuberculosis or sarcoidosis. Moreover, malignant lesions of the skin like basal and squamous cell carcinomas as well as lymphomas, chondrosarcomas, carcinomas of the nasal vestibule, and—on rare occasions—distant metastasis to the facial skin need to be considered [1]. The correct diagnosis is crucial as the uncommon clinical presentation of these cutaneous metastases may lead to the wrong diagnosis and treatment.

## 2. Case Report

**Figure 1:**
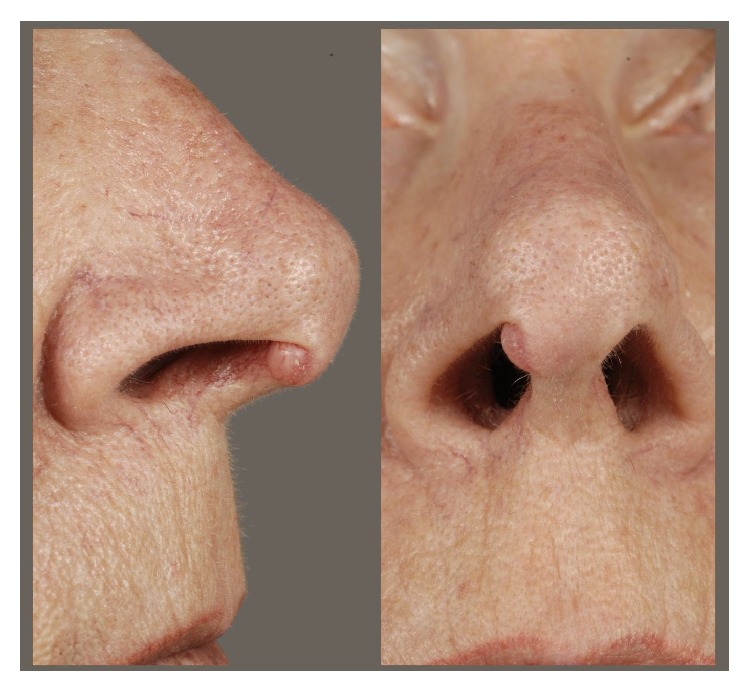
Clinical findings: a tumor measuring about eight millimeters located on the upper border of the right nostril. There was an additional subcutaneous expansion towards the tip of the nose and the columella. The lesion was hemispherical, immobile, sharply demarcated, nonirritated, and painless.

A 59-year-old woman presented to our outpatient clinic complaining of a small indolent tumor on the tip of the nose which was slowly progressing in size. She did not mention any other symptoms (Figure 1).

The clinical examination showed a nodule measuring about eight millimeters which was located on the upper border of the right nostril where the medial crus of the alar cartilage bends to reach the lateral crus. There was an additional subcutaneous expansion towards the tip of the nose and the columella. The lesion was hemispherical, immobile, sharply demarcated, nonirritated, and indolent. Further head and neck examination including laryngopharyngoscopy and cervical sonography did not reveal any other disorder.

The absence of irritation was not consistent with an acute infection. Because of the sharp demarcation, the smooth surface, and the lack of ulceration a primary malignancy was also unlikely. For a better diagnostic classification a histopathological diagnosis was necessary. Using local anesthesia we performed an excision biopsy. Via a small incision, a complete resection of the tumor and its surrounding capsule was possible. Crossing the midline, the lesion expanded into the subcutaneous tissue between the medial walls of the alar cartilages. However, the tumor did not macroscopically infiltrate the surrounding structures. After primary closure and uncomplicated postoperative wound healing the local status was cosmetically unremarkable.

**Figure 2:**
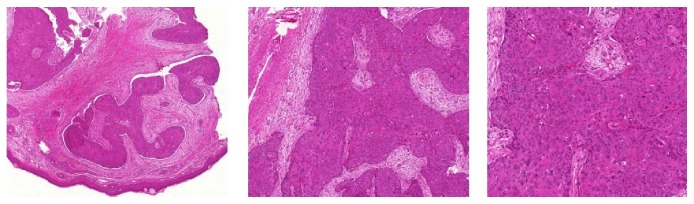
Histopathological examination: fibrous connective tissue partly covered by an intact nondysplastic squamous epithelium. The corial connective tissue is infiltrated by nonkeratinizing squamous cells with pleomorphism of size and nuclei, corresponding to a moderately differentiated squamous cell carcinoma (HE-staining, magnification ×20, ×50, and ×100).

The histopathological examination revealed fibrous connective tissue which was partly covered by an intact nondysplastic squamous epithelium. The corial connective tissue was infiltrated by nonkeratinizing squamous cells with pleomorphism of size and nuclei. These findings corresponded to a moderately differentiated squamous cell carcinoma (Figure 2).

The clinical and histopathological findings, especially the nodular form, the intact epithelium covering the lesion, and the missing infiltration, were not consistent with a primary malignancy of the nasal skin or vestibule. This increased the likelihood of a soft tissue or skin metastasis. During a renewed, targeted anamnesis the patient reported suffering from mild dysphagia and weight loss.

**Figure 3:**
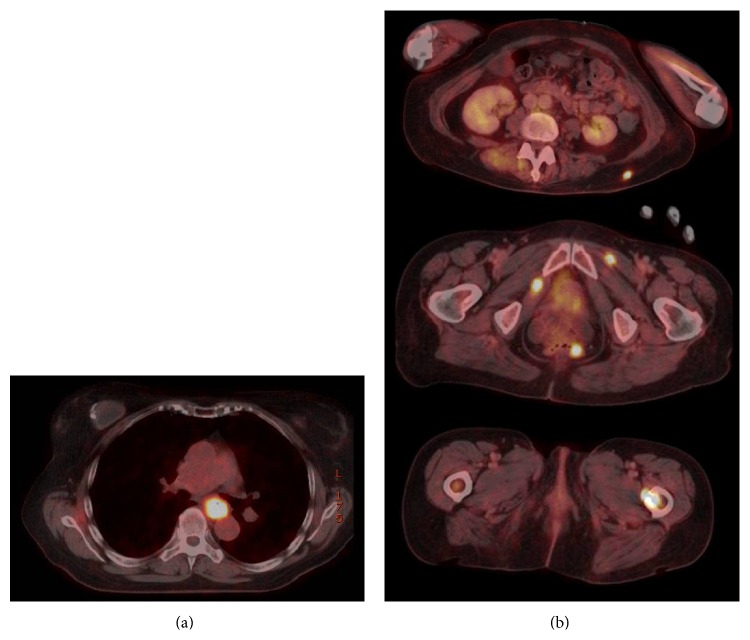
(a) Combined PET-CT scan with 283 Mbq F 18 FDG and computed tomography with iodine biased contrast agent. Area of screening: scull base till proximal femur. It reveals the large soft tissue mass with a significant increase in metabolic activity (SUV max 22.9) which surrounds the dorsal part of the thoracic esophagus. (b) Multiple cutaneous, muscular, and osseous metastases (SUV max 22.9) in the entire range of screening.

After discussing the case in our interdisciplinary tumor conference we performed a PET-CT scan for primary tumor screening. It revealed a large soft tissue mass with a significant increase in metabolic activity which surrounded the dorsal part of the thoracic esophagus. Moreover, in the entire range of screening the scan showed multiple cutaneous, muscular, and osseous metastases, for example, in the right dorsal acetabulum and in the left proximal femur. There were no abnormalities in the head and neck region (Figures 3(a) and 3(b)).

During a flexible gastroesophagoscopy we assessed the extension of the mass in the esophagus. Via a biopsy a squamous cell carcinoma of the esophagus was histologically confirmed.

The treatment was interdisciplinary, including the departments of radiotherapy, oncology, surgery, and palliative care. Over the course of one year the patient received palliative chemoradiotherapy (45,9 Gy, cisplatin, 5-FU) of the primary tumor, resection of several painful soft tissue metastases, palliative radiation of the osseous metastases, and palliative chemotherapy following the FOLFOX-regime. Moreover, stenting of the esophagus was performed. The patient died 18 months after she initially presented to our outpatient clinic.

## 3. Discussion

Hematogenous metastases to the head and neck region are rare and occur predominantly in late tumor states. Metastases seed mainly to the soft tissue, for example, to the maxillary sinus or the main nasal cavity. In decreasing order of frequency, typical primary tumors are renal cell and bronchial carcinomas as well as urogenital and breast cancers. Only 6% of the hematogenous metastases in the head and neck region originate from gastrointestinal malignancies [2]. Distant metastases to the facial skin and the scalp are even more uncommon and are usually a sign of very advanced disease [3].

Infrequently squamous cell carcinomas of the esophagus cause cutaneous metastases: only about one percent of the distant metastases grow in the skin [4, 5]. However, on rare occasions they can be the first sign of a disease. Moreover, they suggest a rapid disease progression and impending generalized metastatic spread [6].

The external nose is rarely affected. So far, metastases to the nasal tip have been reported to arise mainly from underlying renal cell or bronchial carcinomas. The existing literature reveals only three cases where an esophageal cancer was the primary tumor [3, 7, 8]. However, on these occasions the nasal metastasis occurred in a terminal disease stage and was described to be a large, partly necrotic tumor mass (“clown's nose”). Possible reasons for this uncommon localization of a metastasis in esophageal cancer may be iatrogenic tumor cell implantation occurring, for example, from a long-term treatment with a nasogastric tube or lymphatic stasis as seen with big cervical lymph nodes [7]. Both can be ruled out in the presented case. Hematogenous metastasis remains the only possibility.

Uncommon clinical presentation of cutaneous metastases may lead to the wrong diagnosis. Patients have been treated unsuccessfully under the suspected diagnosis of rhinophyma or therapy-resistant infection [9].

Therefore, in the case of a rapidly progressing, initially sharply demarcated and painless nodule of the face and nose a cutaneous metastasis of an unknown primary tumor should be considered in the differential diagnosis. Biopsy for early histological examination should be performed on any suspicious skin lesion. Subsequent positron emission tomography (PET) should be considered in all patients as part of the initial search for the primary tumor [10].

## 4. Conclusion

The differential diagnosis of tumors of the facial skin and the nasal tip includes metastases from an unknown primary tumor. Apart from renal cell and bronchial carcinomas, in rare cases, squamous cell carcinoma of the esophagus needs to be considered. Cutaneous metastases can be the first sign of a malignant disease and have an unfavorable prognostic significance.
